# Conformation-specific detection of calmodulin binding using the unnatural amino acid p-azido-phenylalanine (AzF) as an IR-sensor

**DOI:** 10.1063/1.5053466

**Published:** 2018-11-07

**Authors:** Anne Creon, Inokentijs Josts, Stephan Niebling, Nils Huse, Henning Tidow

**Affiliations:** 1 Department of Chemistry, Institute for Biochemistry and Molecular Biology, University of Hamburg, Martin-Luther-King-Platz 6, D-20146 Hamburg, Germany; 2 Institute for Nanostructure and Solid State Physics, Department of Physics and Center for Free-Electron Laser Science, University of Hamburg, Luruper Chaussee 149, D-22761 Hamburg, Germany; 3 The Hamburg Centre for Ultrafast Imaging, University of Hamburg, Luruper Chaussee 149, D-22761 Hamburg, Germany

## Abstract

Calmodulin (CaM) is a very conserved, ubiquitous, eukaryotic protein that binds four Ca^2+^ ions with high affinity. It acts as a calcium sensor by translating Ca^2+^ signals into cellular processes such as metabolism, inflammation, immune response, memory, and muscle contraction. Calcium binding to CaM leads to conformational changes that enable Ca^2+^/CaM to recognize and bind various target proteins with high affinity. The binding mode and binding partners of CaM are very diverse, and a consensus binding sequence is lacking. Here, we describe an elegant system that allows conformation-specific detection of CaM-binding to its binding partners. We incorporate the unnatural amino acid p-azido-phenylalanine (AzF) in different positions of CaM and follow its unique spectral signature by infrared (IR)-spectroscopy of the azido stretching vibration. Our results suggest that the AzF vibrational probe is sensitive to the chemical environment in different CaM/CaM-binding domain (CaMBD) complexes, which allows differentiating between different binding motifs according to the spectral characteristics of the azido stretching mode. We corroborate our results with a crystal structure of AzF-labelled CaM (CaM108AzF) in complex with a binding peptide from calmodulin-dependent protein kinase IIα identifying the structural basis for the observed IR frequency shifts.

## INTRODUCTION

A variety of important cellular processes are controlled by highly regulated changes in the cytosolic Ca^2+^ concentration.^1^ For many of these effects, the ubiquitous and highly conserved protein calmodulin (CaM) acts as a Ca^2+^ sensor that translates the Ca^2+^ signal into a cellular response.^2^ CaM is exceptionally well conserved across eukaryotes and accounts for >0.1% of the total cellular protein.^3,4^ The structure of CaM comprises two globular domains, each containing two EF-hand type Ca^2+^-binding sites^5,6^ that are connected by a flexible linker helix.^7,8^ Calcium binding leads to conformational changes that enable Ca^2+^/CaM to recognize and bind target proteins with high affinity (K_d_ = 10^−7^ to 10^−11^ M).^2^ The binding of CaM to its binding sites is characterized by enormous structural diversity^9^ with more than 500 natural binding targets known to date.^10,11^ In the most common binding mode, the two CaM domains embrace a short helical segment that often contains dedicated hydrophobic anchor residues that could differ significantly with respect to their spacing (e.g., 1–10, 1–18, or 1–26 binding mode).^9^ Because of the lack of a consensus binding sequence, an easy system for the detection of CaM binding is highly desirable. Recently, binding of calmodulin to peptides has been investigated by IR spectroscopy using an unnatural amino acid (UAA) that was chemically introduced via cyanylated cysteine (-SCN) reporter labels on either CaM or binding peptides.^12,13^

Unnatural amino acids (UAAs) containing cyanide, diazo, and azido groups have absorption bands of the respective CN and NN stretching vibrations that are isolated in frequency from all naturally occurring absorption bands in proteins and water. This absorption is highly responsive in shape and spectral position to the chemical environment (e.g., hydrogen bonding and/or local electric fields), making them sensitive probes of the surrounding structure and dynamics and providing highly localized information on structural dynamics. The spectroscopic behavior of the respective functional groups has been analyzed and gauged by several groups in order to draw a more quantitative conclusion from spectral positions and shapes.^14–20^ Accordingly, vibrational spectroscopy of selectively labelled proteins is becoming an attractive method for investigating structural and dynamic aspects in biomacromolecules. Moreover, the high frequency scale of vibrational spectroscopy of UAAs (corresponding to periods of tens of femtoseconds) enables probing structural dynamics in proteins on the natural (sub-)picosecond timescale of local dynamics.^21–25^

UAAs can be genetically incorporated in defined positions of target proteins using the amber codon suppression technology.^26,27^ This extension of the genetic code beyond the canonical amino acids is achieved by exploiting additional (orthogonal) tRNA/aminoacyl-tRNA synthetase (aaRS) pairs that site-specifically incorporate UAAs in response to (amber) stop codons using the cell's translational machinery. This technology allows labelling *in vivo*, provides precise control about the labelling position, and has been used to incorporate a large number of different UAAs as novel biophysical probes for various purposes.^28–35^ The combination of genetically encoded unnatural amino acids as selective spectroscopic probes with structural studies has also been successfully applied for green fluorescent protein (GFP).^36,37^

p-azido-phenylalanine (AzF) can be genetically incorporated into target proteins using the *E. coli* expression system and evolved tRNA/aaRS pairs from *Methanococcus jannaschii.*^38^ AzF can be used either for crosslinking applications or as a unique IR sensor. Its dipole-active N=N stretching vibration, $\nu_{N_{3}}$, leads to a characteristic IR absorption just above 2100 cm^−1^, a region where native proteins do not display distinct IR absorption bands. The $\nu_{N_{3}}$ mode of AzF exhibits a largely monotonic frequency relationship, which shifts the absorption band maximum to higher frequency with increasing H-bonding strength and, to a lesser extent, with increasing polarity of solvents.^20,22,39^ We note that other UAAs such as cyano-phenylalanine (CnF) seem less ambiguous in this respect.^16^

AzF features an absorption lineshape with a main $\nu_{N_{3}}$ transition and two Fermi resonances,^39^ which can in principle be exploited to gain additional information on coupling of AzF to the protein environment by altered transition strengths and spectral positions of the three respective transitions. The recent study of AzF in isopropanol, D_2_O, and H_2_O by Zhang and co-workers^39^ also shows an increased broadening of the $\nu_{N_{3}}$ band with an increasing vibrational density of states (vDOS) in the mid-infrared frequency range (from isopropanol via D_2_O to H_2_O) and therefore increased fluctuations at higher frequencies. In particular, the DOS of heavy water is very similar to the DOS of H_2_O but rescaled to lower frequency compared due to the heavier deuterium atoms, signaling a correlation of the spectral linewidth of the $\nu_{N_{3}}$ band with the amplitude and frequency of fluctuations in the vicinity of the azido group. Furthermore, local protein fluctuations have reportedly little effect on the homogenous linewidth of the amide I mode, while the inhomogeneous linewidth correlates with solvent accessibility.^40^ These findings may point to a more general behavior of protein fluctuations, leaving homogenous lineshapes largely unaffected.

In this study, we genetically incorporated the unnatural amino acid p-azido-phenylalanine (AzF) in different positions of CaM to exploit the azido stretching mode as a probe of local changes in the chemical environment of AzF in various CaM/CaMBD-complexes by IR-spectroscopy. This system allowed conformation-specific detection of CaM-binding to its binding partners. We further determined the crystal structure of AzF-labelled calmodulin in complex with a peptide from calmodulin-dependent protein kinase IIα, providing a structural explanation of the IR shifts observed upon binding.

## RESULTS AND DISCUSSION

### Selection of labelling positions and CaM-binding peptides as test cases

**FIG. 1.**
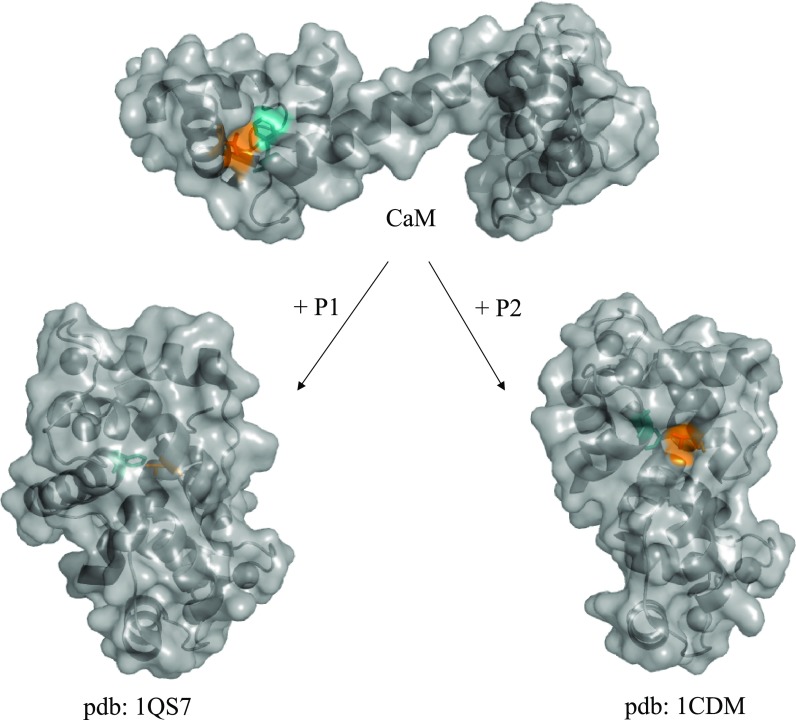
Selection of labelling positions in CaM and conformational changes upon peptide binding. Calmodulin adopts a dumbbell-like conformation and wraps around its target peptide as shown in the complexes with P1 and P2, respectively. Calmodulin is displayed in cartoon/surface representation with Phe92 colored in cyan and Val108 in orange. These positions display different degrees of solvent accessibility in the complexes and were exchanged for AzF for the IR measurements and crystallization.

Ca^2+^-bound calmodulin (Ca^2+^-CaM) has a dumbbell-like structure with two EF-hand containing globular domains connected by a flexible (helical) linker.^5,41^ Upon binding to target peptides, CaM typically wraps around the helical peptides, leading to a dramatic conformational change^9^ (Fig. 1). We selected amino acid positions in CaM which show a different degree of solvent exposure in the free Ca^2+^-CaM structure compared to the structure in complex with CaM-binding peptides. Phe92 was identified as a residue that is solvent exposed in free CaM, while it was always buried in the peptide-bound complex state (Fig. 1). Val108, on the other hand, is also solvent exposed in free CaM but shows a different degree of solvent exposure in various peptide complexes (ranging from exposed to completely buried) (Fig. 1).

**FIG. 2.**
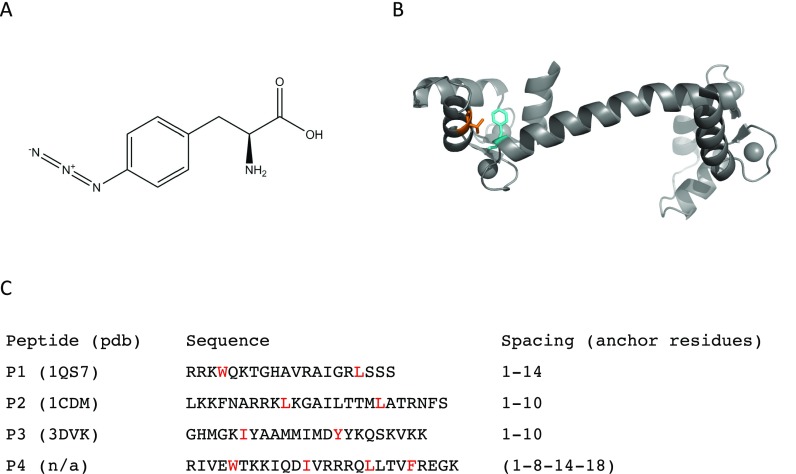
Description of the experimental setup. (a) Chemical structure of AzF. (b) Structure of Ca^2+^/CaM with positions of AzF-incorporation highlighted (Phe92 colored in cyan and Val108 in orange). (c) Peptides used as CaM-binding domains with anchor residues highlighted in red.

These labelling positions in CaM were then combined with several CaM-binding peptides displaying various binding modes in order to probe the large conformational variability of CaM-complexes. We chose four different CaM-binding peptides as test cases, three of them with a known crystal structure and one for which binding was established without a structure being available [Fig. 2(c)]. The selected CaM-binding peptides possess different binding modes characterized by different spacing between hydrophobic anchor residues.^9^ Peptide #1 (P1) is derived from smooth muscle myosin light chain kinase and shows a 1–14 spacing of hydrophobic anchor residues in the crystal structure of its complex with calmodulin (pdb:1QS7). Both P2 (derived from calmodulin-dependent protein kinase IIα/pdb:1CDM) and P3 [derived from voltage-activated calcium channel [Ca(V)/pdb:3DVK] reveal a 1–10 spacing in their CaM-complex structures.^42,43^ In order to apply our findings to previously uncharacterized CaM-binding peptides, we choose P4, a peptide from the TRPM2 Ca^2+^ channel. Based on its sequence, potential hydrophobic anchor residues are located 1-8-14-18 residues apart; however, this anticipated binding mode still requires structural validation. Size-exclusion chromatography (SEC) analysis (supplementary material Figure S1) and isothermal titration calorimetry (ITC) measurements (supplementary material Figure S2) confirmed binding of AzF-labelled CaM variants to the target peptides. All our experiments were performed with Ca^2+^-bound calmodulin (from now denoted CaM for simplicity).

### Peptide binding causes the characteristic shift in CaM92AzF

**FIG. 3.**
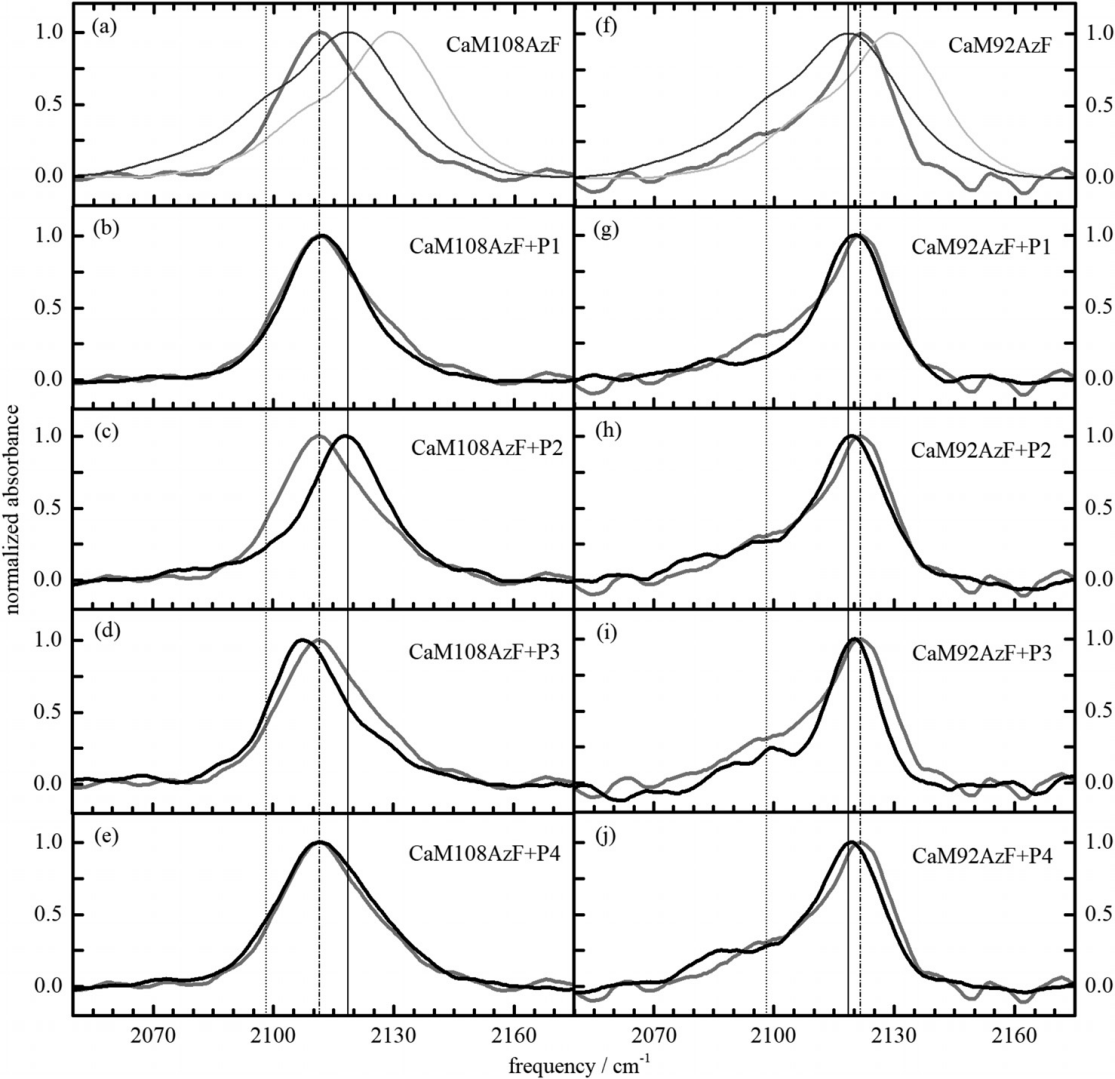
IR spectra of CaM92AzF and CaM108AzF in complex with different binding peptides. The grey spectra in panels (a) and (f) correspond to the AzF-labelled CaM variants without peptide binding. They are also shown in the respective panels below for the CaM proteins in complex with peptides P1–P4 for comparison. Center and centroid values for all measurements are given in Table I. Note that panels (a) and (f) also contain the spectra of the free amino acid AzF in DMSO (black) and in water (light grey). For a better orientation, three vertical lines were drawn. The solid line indicates the main peak of DMSO, the dashed line the shoulder of DMSO, and the dash-dotted line indicates the main peak of the AzF-labelled CaM without peptide binding.

We acquired IR-spectra in a frequency range of 3800–800 cm^−1^ of CaM92AzF alone and in complex with four different CaM-binding peptides (labelled P1–P4) as shown in the right column of panels in Fig. 3. Reference lines help address differences in shape and position of the measured lineshapes: the dotted (∼2098 cm^−1^) and solid (∼2120 cm^−1^) lines mark the pronounced low-frequency Fermi resonance (shoulder) and the main transition (peak) of the $\nu_{N_{3}}$ lineshape of AzF in dimethyl sulfoxide (DMSO), respectively, while the dash-dotted line indicates the spectral position of the uncomplexed CaM92AzF azido stretching band. The spectrum of the $\nu_{N_{3}}$ band of free AzF in aqueous buffer solution is shown in light-gray in the top panels as well.

CaM92AzF features AzF in the cyan position of Fig. 1, which could be conceived as solvent-exposed when CaM92AzF is not complexing a peptide. However, Fig. 3(f) clearly shows that the azido stretching band does not resemble that of free AzF. Instead, it displays a prominent peak at 2121.4 cm^−1^ which is slightly shifted to higher energy compared to the free amino acid in DMSO (a polar but aprotic solvent, i.e., without the capability to donate a hydrogen bond). This observation rules out strong exposure of the azido group to solvent water. Indeed, the $\nu_{N_{3}}$ band shifts to lower frequency by about 1–2 cm^−1^ in all investigated complexes of CaM92AzF+PN (N = 1–4, dash-dotted line in Fig. 3 and Table I), indicating only slight changes in the environment of the azido group, which may be a bit more buried in the complex compared to the free protein. Clearly, an azido group exposed to the aqueous solvent remains absent. High-resolution structures of CaM in complex with the investigated peptides (pdb: 1CDM, 1QS7) indeed confirm that the Phe92 side chain (which is replaced by AzF in our constructs) is deeply buried in the core of the complex and not accessible to the solvent (Fig. 1).

**TABLE I.** Peak positions and line shape analysis for N_3_ bands of AzF-labelled CaM bound to target peptides.

Sample	Centroid (cm^−1^)	Center (cm^−1^)	FWHM (cm^−1^)
CaM108AzF	2114.2	2111.3	26.1
CaM108AzF+P1	2113.2	2112.2	23.3
CaM108AzF+P2	2117.1	2118.0	22.6
CaM108AzF+P3	2109.0	2107.4	22.8
CaM108AzF+P4	2113.5	2111.3	28.9
CaM92AzF	2114.0	2121.4	22.7
CaM92AzF+P1	2113.8	2120.4	19.5
CaM92AzF+P2	2112.9	2119.5	21.1
CaM92AzF+P3	2113.4	2120.4	17.5
CaM92AzF+P4	2111.2	2119.5	22.0

The $\nu_{N_{3}}$ lineshape of all investigated CaM92AzF variants is considerably narrower compared to that of free AzF in DMSO or water, an observation that could either suggest increased fluctuations manifesting in motional narrowing of the spectral lineshape^44–46^ or a polar but not hydrogen-bonded configuration with significantly reduced fluctuations (inferred from a narrower lineshape) and possibly altered intramolecular coupling: The reduced shoulders just below 2100 cm^−1^ and above 2120 cm^−1^ (dotted and solid lines in Fig. 3, respectively) are indicative of a suppression of Fermi resonances in the azido stretching band.^39^ We believe that motional narrowing is the less likely cause for the decreased $\nu_{N_{3}}$ linewidth because the recent 2DIR study of Zhang and co-workers^39^ points toward increased inhomogeneous broadening when changing the solvent from D_2_O for H_2_O (cf. introductory section). Moreover, our lineshape analysis works best for Gaussian rather than Lorentzian lineshapes which suggests that inhomogeneous broadening (and hence a Gaussian-type frequency fluctuation correlation function) dominates the spectral linewidths.

### CaM108AzF can detect different peptide-binding conformations

When substituting AzF in position 108 (CaM108AzF, where Val108 is replaced by AzF), we obtain the infrared spectra as displayed in the left column of panels in Fig. 3. The dotted and solid reference lines are as indicated for the right column. The dash-dotted reference line indicates the peak of the azido stretching band in CaM108AzF.

We observe a characteristic shift of the azido group to lower frequency compared to AzF in DMSO and CaM92AzF. The lineshape of the azido stretching band is also narrower and more symmetric. While the shift to lower frequency may indicate a less polar environment (compared to the polar DMSO molecules), the narrower lineshape again suggests reduced fluctuations of the probed environment and potentially altered mode coupling within the AzF unit by mechanisms already discussed for the CaM92AzF mutation.

In complex with peptides P1 and P4, the lineshape of the azido stretching mode is unaltered, while complexation with peptide P3 (pdb:3DVK) induces an additional softening of this mode by 4 cm^−1^. Most notably, the complex with peptide P2 (pdb:1CDM) yields a shift to higher frequency of almost 7 cm^−1^ [Fig. 3(d) and Table I]. We deduce from this behavior that formation of the complex CaM108AzF+P3 results in reduced polarity around the azido group compared to AzF in DMSO, while the complex CaM108AzF+P2 exhibits an azido environment of similar polarity as provided by DMSO solvation. However, the lineshape of the azido stretching band in the complex with P2 is considerably narrower, lacking the low-frequency Fermi resonance (shoulder) of free AzF in DMSO and water. Note that the Fermi resonance on the high-frequency side of the $\nu_{N_{3}}$ band is still present, i.e., the $\nu_{N_{3}}$ higher-frequency half of the lineshape is similar to the one in DMSO. This observation suggests a different microscopic environment than provided by either DMSO solution or the CaM92AzF variant.

### Crystal structure of the CaM108AzF+P2 complex provides structural explanation for the observed IR frequency shifts

**FIG. 4.**
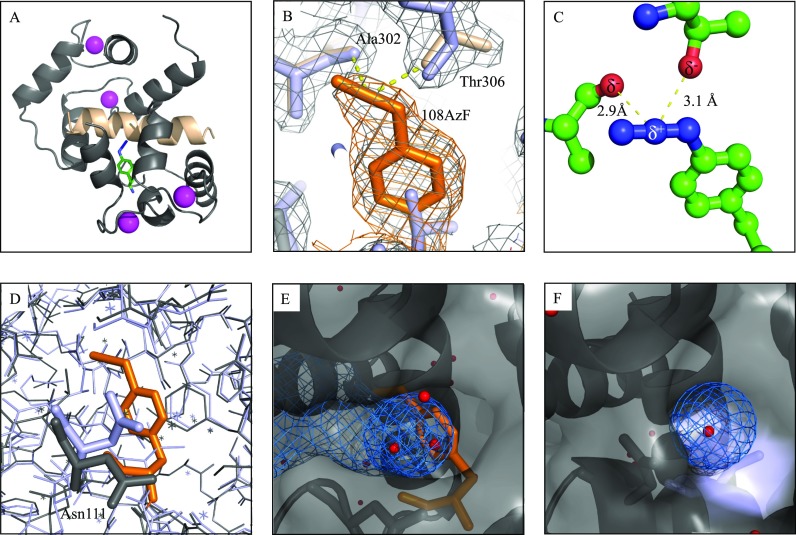
Crystal structure of the CaM108AzF+P2 complex. (a) Overall structure in cartoon representation. CaM is colored grey with 108AzF in green/blue. The peptide is colored in wheat and Ca^2+^ ion in magenta. (b) Polder OMIT map for 108AzF contoured at 3σ illustrating clear electron density for the incorporated amino acid in orange with an additional 2Fo-Fc map shown in grey and the wild-type structure (pdb:1cdm) superimposed in light blue. (c) Details and distances of the weak dipole-dipole interactions between the azido group of 108AzF and carbonyl groups of Ala302 and Thr306 of the binding peptide. (d) Superposition of the CaM108AzF+P2 complex (grey with AzF in orange) with pdb:1cdm (light blue) illustrating the different orientation of Asn111. (e) Representation of the CaM108AzF+P2 complex illustrating the solvent accessibility of 108AzF (orange) due to the orientation change of Asn111. Water molecules are shown in red, and the solvent accessible cavity is shown as blue mesh. (f) Representation of pdb:1cdm at Val108 (light blue) reveals the lack of the solvent accessible cavity in the wild-type complex.

In order to investigate the structural basis for the significant alteration of the azido stretching vibration of AzF-labelled CaM when bound to peptide P2, we determined the crystal structure of this complex. Apart from the region where the AzF label was incorporated, the structure does not reveal any significant structural changes compared to the wild-type protein complex, indicating that the introduction of the label does not alter the overall binding mode (overall RMSD 0.38 Å) [Fig. 4(a)]. Our crystallographic structure refinement shows that the introduced AzF side chain at position 108 points towards the P2 peptide as clearly shown in the electron density (Fig. 4). The azido group is in para-position of AzF108 and interacts with the backbone carbonyl oxygen of Ala302 and the side chain carbonyl oxygen of Thr306 of the bound peptide via dipole-dipole interactions and shows a moderate solvent accessibility [Figs. 4(b) and 4(e)]. The introduction of the AzF label also leads to a conformational change in the Asn111 side chain (compared to the wild-type complex). The position of AzF in the complex structure would sterically clash with the position of Asn111 in the wild-type structure. Thus, the side chain of Asn111 needs to rotate out off the core in the CaM108AzF+P2 complex structure, opening the space between helices H2, H5, and H6 and thus allowing solvent access to the azido group [Figs. 4(d)–4(f)]. We conclude that the dipole-dipole interactions of the azido group with the carbonyl oxygens in AzF108 provide an electrostatic environment that is responsible for the observed shift and change in the lineshape of the azido stretching band. Solvent accessibility may also play a role, but the structural motif in Fig. 4(c) constitutes a fairly rigid (and hence observable) electron density compared to fleeting solvent configurations. We also note that the azido group has a partial positive charge in its center, which appears to interact with one of the lone-pair orbitals in each of the sp^2^-hybridized carbonyl groups as seen in Fig. 4(c). We consider this particular structural motif to be responsible for the characteristically narrower lineshape of the azido stretching band. It may alter intramolecular coupling such as Fermi resonances in this electrostatic configuration.

Our crystallographic structure determination provides unique information on how protein-embedded azido groups interact with the protein backbone and bound substrates. Our method also paves the way for more informed quantum simulations of UAAs and calibration of vibrational probes with respect to local interactions and fluctuations. In general, the azido group has a relatively large transition dipole, making it an attractive probe for the study of protein environments. Its IR spectrum may be difficult to interpret,^24^ but this apparent difficulty also provides additional information on the local environment (e.g., distortion of the structure or charge distribution of the azido group and altered frequency of bending/wagging modes). Our approach of combining Fourier transform infrared (FTIR) spectroscopy and crystallography establishes a microscopic explanation for the observed spectroscopic changes in the infrared spectrum of the azido group with unprecedented detail. The largely monotonic frequency behavior of AzF to proticity and polarity is advantageous but does not allow for unambiguous structural information on the environment of the vibrational marker without simulations and systematic variations of the investigated system. Structural information allows for gauging frequency and lineshape of UAAs. Therefore, providing a crystal structure for particular cases provides important understanding of spectral behavior and ultimately better models for frequency-structure relationships in proteins. We envision that the use of UAAs as vibrational probes of structural dynamics in and out of thermodynamic equilibrium (via time-resolved spectroscopy) will also provide valuable information in more complex environments and will be increasingly employed in biochemical applications.

## METHODS

### Materials

All chemicals were of analytical grade and obtained from Roth (Karlsruhe, Germany) or Sigma-Aldrich (St. Louis, MO), unless otherwise stated. AzF was purchased from Bachem (Switzerland). Peptides were purchased from GL Biochem (Shanghai, China). Peptide identity was confirmed by liquid-chromatography mass spectrometry (LC-MS).

### Expression and purification of AzF-labelled calmodulin

For the incorporation of the unnatural amino acid, we used a modified version of the procedure reported by Young *et al.*^47^ At position F92 and V108, an amber stop codon was introduced by QuikChange site-directed mutagenesis into the CaM sequence. The CaM sequence was cloned into a modified pET28a vector containing a C3 precision site, a kanamycin resistance, and a C-terminal His_6_ tag. The pET28aCaMC3His plasmid was co-transformed with the plasmid pEVOL-pAzF^38^ [a gift from Peter Schultz (Addgene plasmid #31186)] encoding for the evolved tRNA and tRNA-synthetase from *Methanococcus jannaschii* specific for AzF (containing a chloramphenicol resistance and an inducible arabinose promotor) via electroporation into BL21 Gold cells. Overexpression in terrific broth media, supplemented with 25 *μ*g/ml kanamycin and 34 *μ*g/ml chloramphenicol, was performed at 37 °C. At an OD_600_ of 1, 0.02% arabinose, 1 mM p-azido-phenylalanine, 1 mM tryptophan, and 1 mM tyrosine were added. After 45 min, the expression was additionally induced by 1 mM isopropyl *β*-D-1-thiogalactopyranoside (IPTG). The cells were harvested after 4 h and frozen at −20 °C.

For purification, the cells were lysed using an Avestin EmulsiFlex-C3 high-pressure homogenizer. The cell pellet was resuspended in TrisHCl-NaCl-CaCl_2_ (25/150/5 mM) buffer pH 7.4, and 1 *μ*g/ml DNase I and 1 mM MgCl_2_ were added. The cell lysate was cleared by centrifugation, and the supernatant was purified with a Ni-NTA column, equilibrated with TrisHCl-NaCl-CaCl_2_ (25/150/5 mM) buffer pH 7.4. 15 mM imidazole was added to the TrisHCl-NaCl-CaCl_2_ buffer for the wash and 250 mM imidazole for the elution buffer. An evaluation of the purification was done by sodium dodecyl sulfate polyacrylamide gel electrophoresis (SDS-PAGE) (15%), and a dialysis overnight was performed to remove the imidazole. Mass spectrometry analysis confirmed the successful incorporation of the unnatural amino acid AzF. The protein was concentrated using an Amicon Ultra-4, 10 kDa molecular weight cut-off (MWCO) concentrator to a volume of 500 *μ*L. For the protein peptide complex formation, a 1.1 molar excess of peptide was added to the protein solution. All samples were purified via SEC (Superdex 75, 10/300 GL), the identity and purity of the protein containing fraction were checked by SDS-PAGE, and the peak fractions were concentrated and stored at −80 °C until use.

### IR spectroscopy

Attenuated total reflectance-Fourier transform infrared spectroscopy (ATR-FTIR) was performed on a Bruker Vertex 70 spectrometer equipped with a BioATR II unit and a liquid nitrogen-cooled mercury cadmium telluride (MCT) detector. The following protein concentrations were used: CaM92AzF:6.3 mM/CaM92AzF+P1:1.2 mM/CaM92AzF+P2:1.2 mM/CaM92AzF+P3:0.5 mM/CaM92AzF+P4:1.2 mM/CaM108AzF:4.1 mM/CaM108AzF+P1:2.6 mM/CaM108AzF+P2:1.5 mM/CaM108AzF+P3:2.9 mM/CaM108AzF+P4:1.6 mM. The data were collected from 800 to 3800 cm^−1^ with a resolution of 2 cm^−1^ averaging 64 scans. For further data analysis of the spectral region between 2000 and 2200 cm^−1^, OriginPro 2016 was used. All curves were smoothed by Savitzky-Golay interpolation (over 30 points and with second-order polynomials). A baseline correction was performed with a polynomial fit of the fifth order to subtract the slowly varying background below the azido stretching band (Table II). All spectra were normalized to unity to compensate for concentration differences.

**TABLE II.** Region used for the baseline correction of the IR spectra.

Sample	Region (cm^−1^)
CaM108AzF	2080.4–2156.6
CaM108AzF+P1	2062.6–2160.9
CaM108AzF+P2	2075.1–2153.7
CaM108AzF+P3	2063.0–2158.0
CaM108AzF+P4	2075.1–2147.4
CaM92AzF	2095.8–2138.3
CaM92AzF+P1	2069.8–2140.2
CaM92AzF+P2	2072.7–2143.6
CaM92AzF+P3	2067.4–2140.7
CaM92AzF+P4	2104.0–2135.4

### Isothermal titration calorimetry (ITC)

ITC measurements were performed on a MicroCal ITC-200 instrument. The ITC measurements were performed at a cell temperature of 30 °C, with 19 injections and a stirring speed of 750 rpm. The protein with a concentration of 10 *μ*M was dialyzed in 4-(2-hydroxyethyl)-1-piperazineethanesulfonic acid (HEPES)-NaCl-CaCl_2_ (25/150/5 mM) buffer overnight at 4 °C. The peptides with a concentration of 240 *μ*M were dissolved in the same batch of dialysis buffer and centrifuged before use.

### Crystallization and data collection

Initial CaM108AzF+P2 crystallization conditions have been identified using the vapour-diffusion technique in sitting drops in a high-throughput crystallization screen. Then, the most promising hits have been optimized by mixing 1 *μ*l CaM108AzF+P2 solution (20 mg/ml) with 1 *μ*l reservoir solution and equilibrated against reservoir solutions containing 0.2 M lithium chloride, 0.1 M sodium acetate, and 20% (w/v) polyethylene glycol (PEG) 6000 pH 5 at 293 K. The crystals belonged to space group P2_1_ and diffracted to 2.0 Å resolution. Crystals appeared within one week (supplementary material Figure 2). Crystals were mounted and flash-cooled in liquid nitrogen. Diffraction data were collected to a resolution limit of 2.0 Å. Full datasets with an interval of 0.2° were collected at the MX14.1 beamline at BESSY II (Berlin). All datasets were processed with XDS (Kabsch 2010) and merged with AIMLESS (Evans 2006). A summary of the data statistics is given in Table III.

**TABLE III.** Data collection and refinement statistics (molecular replacement). Values in parentheses are for the highest-resolution shell.

	CaM108AzF+P2 (molecular replacement; pdb:6HCS)
Data collection	
Space group	P 1 2_1_ 1
Cell dimensions	
*a*, *b*, *c* (Å)	76.26, 37.21, 121.19
α, β, γ (°)	90, 100.21, 90
Resolution (Å)	43.12–2.0 (2.07–2.0)
*R*_merge_	0.047 (0.035)
*I/*σ*I*	13.62 (3.36)
CC(1/2)	0.99 (0.94)
Completeness (%)	97.59 (98.70)
Redundancy	3.3(3.3)
Refinement	
Resolution (Å)	43.12–2.0 (2.07–2.0)
No. reflections	45027 (4566)
*R*_work_/*R*_free_	0.260/0.294
No. atoms	5239
Protein	4996
Water	243
*B*-factors (Å^2^)	
Protein	49.95
Ligand/ion	55.03
Water	46.28
R.M.S. deviations	
Bond lengths (Å)	0.008
Bond angles (°)	1.15

### Structure determination, refinement, and analysis

The structure was solved by molecular replacement using PHASER^48^ using pdb:1cdm^43^ as a search model. Subsequent rounds of manual building using COOT^49^ and refinement using phenix.refine^50^ allowed complete model building and revealed clear density for the incorporated AzF at position 108 (Fig. 4). The final models yielded crystallographic *R*-factors of 0.26/0.29 (R_work_/R_free_) (see Table III for crystallographic details). The models were validated using MolProbity.^51^ Evaluation of the Ramachandran plot showed all residues in allowed regions (98% in favoured regions). Polder OMIT maps^52^ were used to visualize AzF density. All figures were prepared using PyMOL^53^ and the CAVER plugin.^54^ The data are deposited in the Protein Data Bank with pdb code 6HCS.

## SUPPLEMENTARY MATERIAL

See supplementary material for SDS-PAGE, crystal images, and ITC data of the CaM108AzF+P2 complex.
